# Earthquake Preparedness in Iranian Hospitals: A Systematic Review and Meta-Analysis

**DOI:** 10.30476/BEAT.2021.86968

**Published:** 2021-01

**Authors:** Daem Roshani, Aram Karimian

**Affiliations:** 1 *Social Determinants of Health Research Center, Research Institute for Health Development, Kurdistan University of Medical Sciences, Sanandaj, Iran*; 2 *Department of Emergency Medical Sciences, School of Paramedical Sciences, Kurdistan University of Medical Sciences, Sanandaj, Iran*

**Keywords:** Hospitals, Earthquakes, Iran

## Abstract

**Objective:**

To assess the preparedness of Iranian hospitals against earthquake.

**Methods:**

In this systematic review, a query was carried out on PubMed, Scopus, Web of Sciences, Sid, Irandoc, Google scholar, and Magiran databases for articles published between 2000-2019. Statistical analysis was performed using Chi-square and I2 tests at a confidence interval of 95%. Finally, out of 7458 studies, 10 related articles were analyzed.

**Results:**

As evidenced by the obtained results, the highest readiness was obtained at 0.709 (95% CI: 0.49-0.88) in “disaster plan”, while the lowest readiness was reported at 0.455 (95% CI: 0.23-0.68) in “structural safety”. The overall earthquake preparedness of these hospitals was calculated at 0.47 (95% CI: 0.37-0.56).

**Conclusion:**

The results of the present study pointed to the moderate level of earthquake preparedness in Iranian hospitals. This finding highlights the necessity of a training plan and implementation of a national program in all hospitals of the country to increase earthquake preparedness.

## Introduction

Large earthquakes are one of the most destructive natural disasters, often resulting in massive casualties and high mortality [1]. As reported by previously conducted studies, the majority of health care facilities were either destroyed or out of function in earthquake-prone areas. Moreover, only a small number of injured patients are able to reach field hospitals, which are an essential component of supportive operations usually set up after 24 hours or later [2]. even if the hospital is not demolished during the earthquake, hospitalization of the injured imposes a heavy burden on these centers [3, 4], which not only worsens the situation for patients and hospital staff but also affects community health. Therefore, it is necessary to maintain hospital activity and critical services during a crisis [5, 6].

Preparedness is defined as activities set up to build a mechanism for rapid responses to limit the risks and effects of disasters [7] and is regarded as the most important step in the disaster response cycle [8, 9]. The World Health Organization (WHO) always puts emphasis on acquiring the three components of disaster preparedness, including structural, non-structural, and functional preparedness [10]. Earthquake preparedness is of paramount importance in hospitals since they are the first place the injured are referred to [11]. Hospitals should continue health care provision at the time of disasters and this requires preparation [12, 13]. Lack of prevention and preparedness for an effective response can lead to a painful tragedy since hospitals are considered the most important centers for disaster relief and are among the first organizations that get affected [14, 15]. 

Considering the high incidence of earthquakes almost everywhere in the world, it is important to address the issue of earthquakes and prepare the health care system to provide care in critical situations. The present study aimed to systematically assess the preparedness of Iranian hospitals against earthquakes to help integrate all the key components (10 variables) of preparedness and give the reader a general view of these components and ways to upgrade them. It is hoped that the findings of the current study will assist the managers and authorities of hospitals and medical centers in increasing earthquake preparedness.

## Materials and Methods

In the present systematic review and meta-analysis, the relevant studies were selected, and the results were reported based on the PRISMA guidelines [16].

Precise Definition of the Study Question and Identification of Its Components 

At this stage, a detailed definition was provided for the following: participants in the baseline study, type of intervention, comparison group, outcome, type of baseline studies, as well as the time and place of review. The appropriate keywords were selected according to MESH (Medical Subject Headings) and EMTREE. The queries were carried out with high sensitivity to ensure the identification of all preliminary studies. The search strategy for each of the databases is explained in the following. The current research was performed using some keywords, including “hospitals”, “earthquake”,” disaster”, “preparedness” and “Iran”. The Persian equivalent of these terms and all possible combinations in the Persian national databases were also searched. 

Search for Basic Studies 

The query was carried out on both International databases (including PubMed, Google Scholar, Web of Science, Science Direct, and Scopus) and Persian national databases [namely SID (Scientific Information Database), Medlib (Iranian Medical Library), Iran Medex (articles published in Iran Biomedical Journals), Magiran and IranDoc] for published articles on the preparedness of Iranian hospitals. Duplicates were removed after searching and logging the findings into the Mendeley software to find relevant articles. 

Inclusion and Exclusion Criteria for Study and Selection Process 

Two authors independently performed all the stages of study selection, separation by title, the abstract or full text of articles, and data extraction. Any disagreement between the two authors was resolved by asking the opinion of the third author. The inclusion criteria entailed all studies published as an original article. On the other hand, the exclusion criteria were as follows: 1) studies without original research (reviews, editorials, non-research letters), 2) poor-quality studies that did not report a good index of association between hospital preparedness and earthquake, 3) insufficient data, and 4) studies published in Persian without an English abstract. Primarily, 7433 articles were retrieved, and 24 papers were excluded due to duplication. Out of the remaining 7,401 articles, 7,309 papers were excluded for the following reasons: irrelevancy, non-original study, pilot study, and repetition of studies. Those articles indexed in several databases were regarded as one. Out of the remaining 90 articles, 69 papers were removed due to the absence of study design specification and insufficient information about component data. The other 21 articles were removed since they had qualitative synthesis. 

In the event of incomplete or ambiguous data, the author(s) were requested for more detailed information via email. Initially, the authors read the titles of articles and selected the relevant ones. At this stage, they were sensitive and reviewed the full text of the articles related to the STROBE and MOOSE or NEWCASTLE - OTTAWA (NOS) tools in terms of quality. 

Data Extraction 

Finally, 10 related articles were reviewed. The necessary data were accurately acquired via a data extraction form on the basis of title, year of publication, type of study, region (province), and sample size; subsequently, they were entered the pre-prepared forms. 

Synthesizing and Integration of Data and Meta-analysis 

Two researchers independently reviewed the collected articles based on inclusion and exclusion criteria, and in case of any disagreement, another author examined them. Finally, the collected information was entered into the software. The prevalence of each component was reported with a 95% confidence interval. Weighted averages were calculated for each study based on sample size and variance. Two models of static effect (DerSimonian and Laird method) and random effect (Mantel and Haenszel method) were used according to the results of the heterogeneity of the studies. Statistical analysis was performed using the Chi-square test and I2 at a 95% confidence level. In addition to statistical tests, cumulative and Galbrais graphs were also used. In the accumulative graph, the amount of heterogeneity was visualized to find outlier data. Begg’s funnel plot and Egger’s linear regression test were used for assessing publication bias. All the statistical analyses were performed using Stats Direct software using a two-tailed test. A *p*-value less than 0.05 was considered statistically significant. 

## Results

**Fig. 1:**
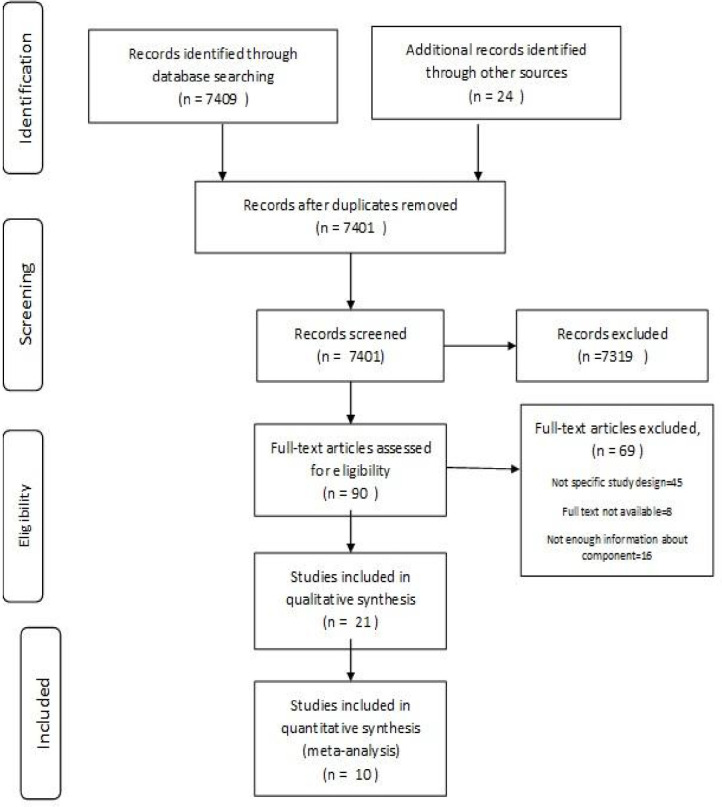
PRISMA flow diagram. PRISMA: Preferred Reporting Items for Systematic Reviews and Meta-Analysis

In the present study, out of 7458 studies,10 articles were reviewed (Figure 1). The results of the percentages of preparedness components by year and the number of hospitals involved in the studies are presented in Table 1. The highest preparedness was obtained at 0.955 for disaster plan management, while the lowest was reported as 0.14 for hospital evacuation (Table 1). Table 2 illustrates the percentage of merged components with 95% confidence intervals. More than half of the components were less than 50%. The highest preparedness was obtained at 0.709 (95% CI: 0.49-0.88) for disaster plan, while the lowest preparedness was reported as 0.455 (95% CI: 0.23-0.68) for construction mitigation. The overall earthquake preparedness of these hospitals was calculated at 0.47 (95% CI: 0.37-0.56) (Table 2).

**Table 1:** Distribution of Eleven Preparation Percentages of Hospital Preparedness against Earthquake by Author Name, Year and Number of Hospitals

**Author**	**Year**	**Sample**	**Non-Structural Vulnerabilities**	**Disaster plan management**	**Support for critical services**	**Medical and nonmedical equipment**	**Hospital environmental health proceedings**	**Hospital curriculum program**	**Construction mitigation**	**Safety of equipment and hazardous materials**	**Hospital evacuation**	**Human resources**	**Overall preparedness**
Arab (4)	2008	15			0.6118	0.5511	0.4708	0.3741	0.45	0.4002	0.3754	0.5669	0.4954
Daneshmandi (9)	2010	30		0.80		0.702		0.686			0.488	0.443	0.544
mohammadi yeganeh (13)	2011	7	0.5457			0.29					0.14		
Hekmatkhah (20)	2012	10		0.4417	0.4118	0.20	0.437	0.528	0.625	0.2138	0.1684		0.207
Rabeian (14)	2013	4		0.8725	0.72	0.55	0.40	0.4125	0.28	0.495	0.415		0.5181
Afkar (21)	2013	19		0.43	0.43	0.26	0.43	0.64	0.83	0.66	0.59		
Asadzadeh (15)	2014	22											0.338
hosseini shokouh (19)	2014	15		0.955	0.972	0.791	0.833	0.933	0.566	0.812	0.912		0.855
Heidaranlu(10)	2016	8	0.6375										
Abedi (22)	2017	4		0.8908	0.67	0.39	0.38	0.25	0.26	0.33	0.33		0.5887

**Table 2:** Integrated percentages of hospital preparedness components against earthquake with 95% confidence interval

**95% CI (exact)**		**Pooled proportion**	***p*** ** value**	**I** ^2^	**Q**	**Total sample**	**Component**
**Upper**	**Lower**						
0.688	0.231	0.455	0.0013	74.8%	19.837	67	Construction mitigation
0.707	0.346	0.528	0.0368	57.8%	11.857	67	Safety of equipment and hazardous materials
0.665	0.317	0.490	0.0017	69.6%	23.041	104	Hospital evacuation and field treatment
0.650	0.326	0.487	0.0059	64.7%	19.854	104	Necessary medical and nonmedical equipment
0.697	0.267	0.480	0.004	71%	17.255	67	Hospital environmental health proceedings
0.736	0.323	0.533	0.0002	77.3%	26.472	97	Hospital curriculum program
0.881	0.499	0.709	0.0022	73.3%	18.734	82	Disaster plan management
0.875	0.406	0.662	0.0003	78.8%	23.541	67	Support for critical services
0.804	0.353	0.588	0.8382	*%	0.041	15	Non-Structural Vulnerabilities
0.619	0.340	0.479	0.412	*%	0.673	46	Human resource
0.564	0.376	0.470	0.4135	1.4%	6.087	100	Overall preparedness

**Fig 2:**
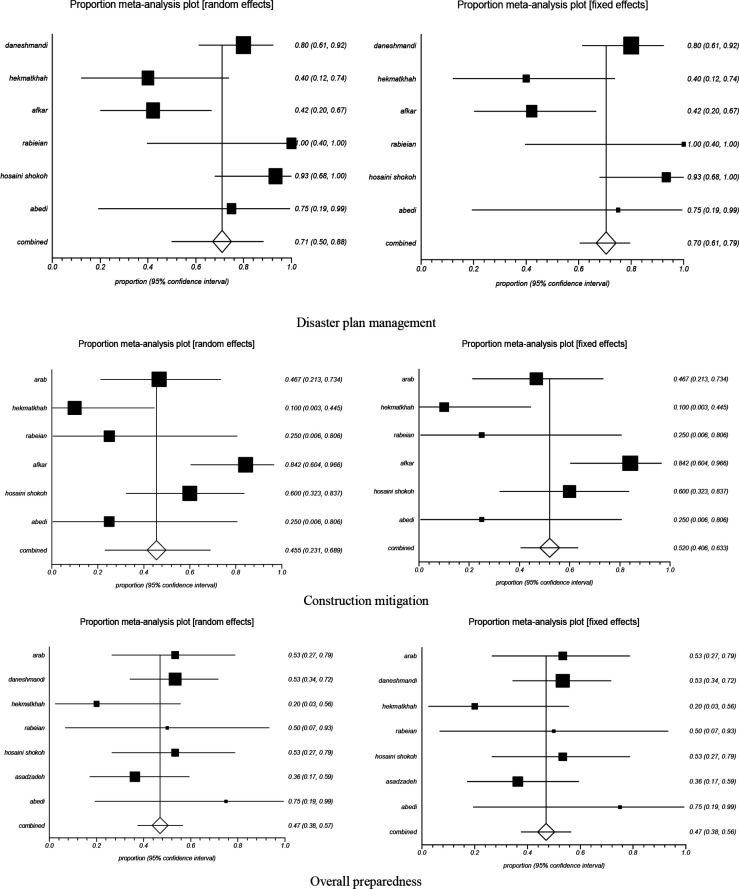
Forest plot with 95%CI for pooled proportion of Disaster plan management, Construction mitigation and overall preparedness

**Fig 3:**
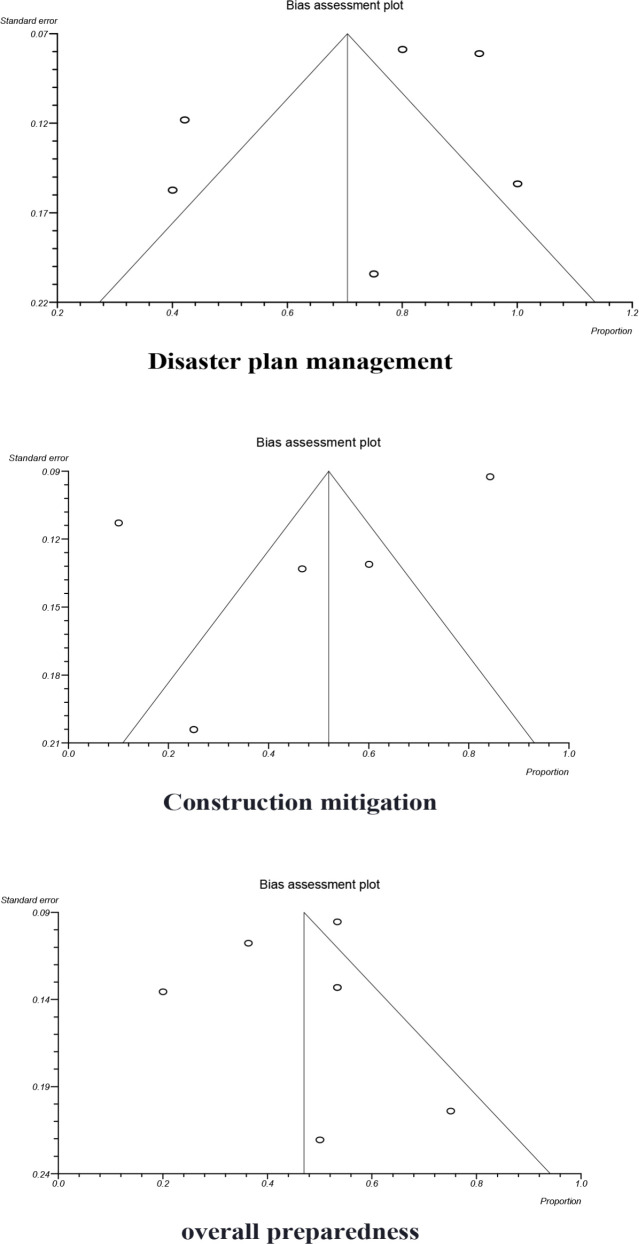
Funnel plot detecting biases in the identification and selection of studies

The Forest Chart of these three components is based on Random and Fixed models in Figure 2. Except for the two components of Human Resource and Overall Preparedness, the results of Q and I^2^ pointed to the superiority of the Random Model over the Fixed one. Begg funnel plot and Egger’s linear regression test was used for the evaluation of publication bias. The results of this test (*p*>0.05) and graphs indicated no publication bias among studies (Figure 3). Furthermore, the sensitivity analysis was performed to evaluate the effect of the inclusion of each study on this meta-analysis. The results of the meta-analysis demonstrated the complete similarity among the merged percentages.

In the majority of studies (n=5), data about the preparedness of hospitals against earthquakes were collected via the earthquake preparedness assessment checklist in eight planning dimensions. This checklist encompasses the following issues: 1) safety of equipment and hazardous materials, 2) mitigation of construction, 3) hospital evacuation and field treatment, 4) necessary medical and nonmedical equipment and consumable goods, 5) hospital environmental health proceedings, 6) hospital curriculum, 7) disaster plan management, and 8) support planning of critical services. In this 123-item questionnaire, according to the earned scores, the situation of earthquake preparedness in each planning dimensions was categorized into three groups: A) weak (0-<50%), B) moderate (50%-<75%), and C) good (75%-100%). In other studies, researcher-made questionnaires were used.

## Discussion

The present study aimed to evaluate earthquake preparedness in Iranian hospitals. In this systematic review, we analyzed the overall preparedness of 134 Iranian hospitals for 10 components against earthquakes. The results pointed out that the earthquake preparedness in Iranian hospitals is at a moderate level (pooled proportion=0.470). In a systematic review performed by Kazemzadeh *et al*., [17] the preparedness of 51 Iranian hospitals against disasters was assessed to be at a moderate level. The lack of preparedness programs and resources were recognized as the major causes of their weakness. In a survey conducted on 425 Iranian hospitals, Ardalan *et al*., [18] stated that hospital preparedness is at a moderate level; nonetheless, this readiness has been improving in recent years which can be ascribed to investment in crisis management and promotion of HIS programs. 

In the present study, the highest level of preparedness is related to the component of Disaster plan management (pooled proportion=0.709). This program identifies policies, protocols, and ways in which hospitals respond to the crisis. In the same context, in their study, Rabeian *et al*., [14] reported that 25.87% of hospitals were well prepared. The existence of a crisis committee, a clear job description of committee members, and collaboration with other supporting organizations, such as the Red Crescent and military services, are among the strengths of hospitals. 

In addition, based on the results of the current study, the lowest level of earthquake preparedness was related to the dimension of structural safety (pooled proportion=0.455). Hosseini Shokouh *et al*., [19] explained that although the safety of architectural components does not directly affect hospital performance, their demolition at the time of the earthquake can cause serious disruption to hospital functions. They added that hospitals are easily demolished in earthquakes for the following reasons: the erosion of hospital buildings, insufficient supervision of relevant agencies during construction, failure to identify hospital building vulnerabilities, and lack of budget allocation to strengthen and rebuild these centers.

Concerning the safety of equipment and hazardous materials, the findings of the present study pointed to the moderate level of preparedness in Iranian hospitals in this dimension (pooled proportion=0.528). Along the same lines, in their study, Hekmatkhah *et al*., [20] attributed this result to the following reasons: non-identification of hazardous materials in the departments, the lack of protection measures for personnel in the event of a common post-earthquake infectious disease, and the absence of specific treatment programs for personnel exposed to radioactive materials, as well as chemical and biological contaminants.

In terms of hospital evacuation and field treatment, Iranian hospitals have been reported to be at a moderate level. In their study, Afkar *et al*., [21] rated hospital readiness in this area as moderate. They revealed that the development of plans for emergency evacuation and treatment in open spaces, identifying evacuation teams, and emergency exit pathways are among the notable strengths of hospitals. Nevertheless, the lack of safety equipment in the wards, inadequate space for out-patient treatment, as well as insufficient tents and removable beds for the field hospital have been cited as the major weaknesses of the studied hospitals.

Regarding support for critical services, as displayed in Table 2, Iranian hospitals are in a favorable position (pooled proportion=0.662). This ensures that in the event of an earthquake, hospitals can continue to operate and provide vital services. Abedi *et al*., [22] attributed this finding to the existence of an uninterrupted power generator in most hospitals, provision of necessary measures to preserve care for hospitalized patients in intensive care units, and provision of life-saving equipment.

Non-structural vulnerabilities and human resources are other important aspects of earthquake preparedness in Iranian hospitals which have been scarcely studied (two articles). Non-structural elements generally include mechanical and telecommunication, while electrical components encompass water supply, heating and cooling systems, fire detection, and containment systems. In this regard, Iranian hospitals were moderately prepared (pooled proportion=0.588). In terms of human resources, the preparedness of Iranian hospitals is at a moderate level. Insufficient attention to the needs of staff and their families in times of earthquake and lack of proper staff training can be among the weaknesses of hospitals in this regard. From this perspective, the notable strengths of hospitals included completing and updating the staff contact list, assessing and monitoring staff presence and absence, and prioritizing personnel need [22].

Furthermore, studied hospitals were also at a moderate level regarding the necessary medical and nonmedical equipment, environmental hospital health planning, and hospital curriculum. In the field of essential supplies, Afkar *et al*., [21] identified the shortage of extra beds, trolleys, and wheelchairs, lack of hospitalized pharmaceutical equipment, lack of blood bank reserves, and periodic review of consumer medications as weaknesses of studied hospitals. In terms of hospital training, Hosseini Shokouh *et al*., [19] stated that there were training courses in crisis management in all the studied hospitals. On the contrary, in the field of rescue and relief, one of the major weaknesses of the studied hospitals was the failure to train patients on rescue techniques to respond in the event of an earthquake.

In the dimension of planning environmental health measures, the studied hospitals demonstrated a low level of preparedness. This unsatisfactory level can be attributed to the failure to plan for food control, disinfect different parts of the hospital, construct temporary facilities, plan for proper collection of wastewater, and plan for providing water and hygienic materials in different parts after the earthquake. In addition, there is an absence of planning for chemical and biological quality control of hospital water, as well as the lack of a potable water treatment system for disinfection of water after the earthquake [20, 22].

The assessment of earthquake preparedness in Iranian hospitals demonstrated that although many components have been studied, some components, such as surge capacity, disaster recovery, triage, communication, safety, and performance of earthquake maneuvers have been scarcely evaluated and require more studies in these areas. There is also a need to focus more on hospital coordination and connection with other relief groups, such as emergency medical services, the Red Crescent, and the fire department. As a final note, one of the main missed elements in earthquake preparedness is the failure to recruit, train, and deploy volunteers during the earthquake which requires careful planning by the authorities.

As evidenced by the results of the present study, the earthquake preparedness in Iranian hospitals was reported to be at a moderate level. This finding highlights the necessity of training and implementation of a national program in all hospitals of the country to increase earthquake preparedness. The mere development of earthquake prevention programs is not effective, rather it should be implemented and practiced regularly and continuously. Furthermore, it was suggested to increase structural and nonstructural safety, provide the necessary medical equipment, establish the appropriate communication infrastructure, continue staff training courses, and promote coordination and collaboration with other relief teams to increase hospital readiness. 
